# Spousal Support and Strain in Relation to Concerns About Aging: The Serial Mediating Roles of Life Satisfaction and Attitudes Toward Own Aging

**DOI:** 10.21203/rs.3.rs-9833319/v1

**Published:** 2026-06-05

**Authors:** Shuming Zhang, Mengdi Wu, Peng Yao, Weihan Qiao, Feng Tian, Fengbo Liu

**Affiliations:** Zhengzhou University of Light Industry; Zhengzhou University of Light Industry; Zhengzhou University; Zhengzhou University of Light Industry; Zhengzhou University of Light Industry; Zhengzhou University of Light Industry

**Keywords:** Spousal support, Spousal strain, Life satisfaction, Attitude toward own aging, Aging concerns, Older adults

## Abstract

**Objectives:**

Concerns about aging (CA) refers to fears and apprehensions related to aging, which may contribute to adverse psychological and physical outcomes in later life. The present study aimed to examine the associations between spousal relationship quality and CA among older adults.

**Methods:**

Drawing on data from the 2022 wave of the Health and Retirement Study (HRS), a nationally representative survey of older adults in the United States, this study examined the associations between spousal relationship quality and concerns about aging using structural equation modeling.

**Results:**

The mediation analysis indicated that both spousal support (SPS) and spousal strain (SPN) were significantly related to CA through significant total and indirect effects (all β ≥ 0.071, 95% CIs excluding zero), although the direct effects were not significant. Indirect pathways showed that life satisfaction (LS) and attitudes toward own aging (ATOA) jointly mediated the effect of SPS on CA, whereas the serial mediation pathway via LS and ATOA was significant for SPN, while no significant single mediation effect was observed.

**Discussion:**

Distinct emotional–cognitive mechanisms were identified underlying the associations of spousal support and strain with concerns about aging. These findings highlight the role of marital dynamics in shaping aging concerns in later life.

## Introduction

Health challenges arising from population aging and the increasing prevalence of chronic physical and mental health conditions have become two of the most pressing global concerns. According to the World Health Organization (WHO), the global population aged 60 years and older surpassed 1.1 billion in 2023 and is projected to reach 1.4 billion by 2030 (World Health Organization, 2025). This demographic shift highlights the continuing trend of population aging. The burden of physical and mental health conditions among older adults, including cardiovascular disease and cognitive impairment, is expected to increase, placing substantial pressure on global health systems (Hotez et al., 2014). Additionally, rising healthcare expenditures associated with population aging may have widespread implications for economic growth, public health systems, and social welfare (De Meijer et al., 2013). Therefore, identifying effective strategies to promote healthy and positive aging among older adults has become increasingly important.

At the individual level, advancing age brings multiple challenges that affect older adults across physical, psychological, and social dimensions. One investigation showed that about one in four older adults reported memory loss, one in five reported living with a severe illness and frequently experiencing sadness or depression, and one in ten reported feeling unneeded by others (Cohn, 2009). Preventing and addressing these adverse conditions has become an increasingly important concern. Previous research has indicated that depression commonly occurs in older adults and is strongly correlated with physical health problems, including cardiovascular disease, hypertension, diabetes, and other chronic conditions (Penninx et al., 1999). Moreover, various forms of psychological stress, including depression and anxiety, have been shown to accelerate the progression of chronic disease, likely through underlying physiological pathways (Hamer et al., 2008). A wide range of factors may influence depression and related mental health outcomes. Among these factors, worry has been identified as a particularly salient antecedent and has demonstrated a robust association with depressive symptoms (Vîslă et al., 2022). Concerns about aging (CA) refer to worries and apprehensions related to aging and later-life decline, including concerns about physical deterioration, cognitive impairment, dependency, and loss of autonomy(Lasher & Faulkender, 1993). Unlike general psychological distress, CA reflects aging-specific perceptions and anticipations that may shape health and well-being in later life(Sargent-Cox et al., 2014). Given these implications, research on CA is particularly important, as CA represents a critical factor linked to physical health, cognitive decline, economic security, and social support among older adults.

House et al. (1988) emphasized that social support, especially close support, plays a critical role in alleviating stress, fostering mental well-being, and reducing the adverse consequences associated with stress. Subsequent research has established spousal relationships as key determinants of health, shown to bolster resilience (Coyne & DeLongis, 1986), improve immune functioning, and lower the risk of disease (Kiecolt-Glaser & Newton, 2001). Recent research suggests that spousal relationships are not only central to individual health but also constitute crucial determinants of health and well-being among older adults (Umberson & Karas Montez, 2010). Accordingly, it is reasonable to expect an association between spousal relationships and aging-related concerns. Moreover, examining the mediating processes underlying this relationship may yield important insights into its underlying mechanisms and identify additional factors associated with CA in older adults.

### Spousal support and strain on concerns about aging

Empirical evidence consistently demonstrates a strong link between spousal relationship quality and well-being. Mäki et al. (2025) found that stable spousal relationships serve as a crucial determinant of mental well-being among older adults. However, how spousal relationships confer benefits remains a key question, and different strands of research have identified multiple perspectives to explain the underlying mechanisms and pathways. Wilson and Novak (2022), through research on spousal behaviour, demonstrated that communal behaviour can significantly predict health levels and that spousal relationships are influenced by behavioral mechanisms. Moreover, high-quality relationships lay the groundwork for effective health behaviour intervention, as they foster behavioural change and subsequently improve health outcomes (Gouin & Dymarski, 2024). Collectively, the evidence from these studies indicates a robust association between spousal relationship quality and a range of health outcomes. For older adults, perceived spousal support (SPS) corresponds closely with received support and has been shown to significantly correlate with biological markers of aging, including inflammatory responses, immune functioning, and other related physiological parameters (Wilson et al., 2021). Evidence from Korean samples suggests that higher marital satisfaction enhances levels of social participation, which may serve as an important mechanism underlying this association (Kim, 2023). In long-term spousal relationships, such as marriages lasting more than 40 years, longitudinal studies identified that marital stability is associated with better mental health among older adults(Margelisch et al., 2017). Whether considering spousal relationship quality or marital stability, there is a clear pathway from SPS to mental health, which provides a theoretical basis for expecting associations with concerns about aging among older adults.

From another perspective, spousal strain (SPN) may lead to adverse outcomes. Previous research has suggested that negative aspects of spousal relationships are significantly associated with physical functioning, including chewing ability, vision, and hearing ability (Kim & Kwon, 2024). Such adverse outcomes may contribute to accelerated aging processes, providing further evidence that negative aspects of spousal relationships constitute strong predictors of later-life trajectories (Hwang et al., 2025). Accordingly, we hypothesize that SPN is associated with heightened concerns about aging among older adults, potentially reflecting distinct pathways from those of SPS..

H1: SPS is negatively related to CA among older adults.

H1a: SPN is positively related to CA among older adults.

### The mediating role of life satisfaction

Life satisfaction (LS) is defined as an individual’s overall cognitive and subjective appraisal of their life circumstances (Diener et al., 1985), representing the degree to which individuals evaluate their lives as satisfactory based on personal standards and values (Diener et al., 1999). LS is often regarded as a core component of subjective well-being (SWB) and, in some domains, is referred to as cognitive well-being. To date, LS has been established as a reliable indicator reflecting the comparison between one’s ideal life and actual circumstances (Vittersø, 2025). LS has been found to be significantly associated with mental health, as individuals with higher levels of LS tend to report lower levels of stress, depression, and loneliness (Diener & Seligman, 2002). For older adults, a worldwide review indicated that higher levels of LS can predict lower depression risk, self-reported health, and longer life expectancy (Steptoe et al., 2015). However, social relationships play an important role in shaping LS, emphasizing the critical link between social connectedness and well-being. A longitudinal study found that SPS and SPN were significantly associated with LS among older adults and used trajectory analyses to demonstrate that marital quality serves as a stable predictor of subjective well-being over time (Carr et al., 2016). LS plays a key role in psychological processes and may mediate the relationship between spousal relationship quality and aging-related concerns. Accordingly, we propose the following hypotheses in this study:

H2: LS mediates the relationship between SPS and CA.

H2b: LS mediates the relationship between SPN and CA.

### The mediating role of attitude toward own aging

Lawton (1975) proposed the construct of Attitude Toward Own Aging (ATOA) as a central dimension for evaluating mental adaptation to the aging process, and demonstrated significant associations between ATOA, LS, and depression. Levy et al. (2002) found that high levels of self-perceptions of aging (SPA) were significantly associated with lower levels of depression, and predicted greater life expectancy. Moreover, the Stereotype Embodiment Theory (SET) suggests that negative ATOA derives from internalized age stereotypes, which manifest as depression, stress and helpless, and can stimulate physiological responses and affect cognitive functioning (Levy, 2009). Longitudinal research indicated that ATOA predicts trajectories of mental health over time, and negative ATOA was significantly linked to greater increases in adverse psychological outcomes over the long term (Sargent-Cox et al., 2012). From a psychological perspective, attitudes are conceptualized as multidimensional value orientations that may give rise to concerns and fears involving both affective and cognitive components. CA plays a key role in mental health among older adults, and elevated CA may contribute to lower levels of LS and mental health problems such as depression. An association may be expected between CA and ATOA, given that ATOA constitutes a psychological dimension reflecting individuals’ cognitive and emotional evaluations of their aging process. Because perceptions of aging are often shaped by close interpersonal environments, particularly spousal interactions, emotional support and strain within marriage may profoundly influence both LS and ATOA. Positive marital relationships tend to enhance emotional well-being and foster adaptive attitudes toward aging, whereas negative or stressful relationships may reinforce aging-related fears and negative self-perceptions. Marital quality is also significantly associated with mental health. In this regard, we propose the following hypothesis:

H3: ATOA mediates the relationship between SPS and CA.

H3c: ATOA mediates the relationship between SPN and CA.

### The serial mediating role of LS and ATOA

Hayes (2017) revealed that multiple mediators can transmit the effect of an independent variable on a dependent variable within a causal sequence, and further emphasized that understanding such indirect pathways helps researchers uncover how and why effects occur, rather than merely confirming that they exist. Moreover, he argued that mediation should be viewed as evidence of underlying mechanisms, highlighting the importance of theory-driven modeling and conditional process analysis in psychological research. We propose potentially complex mechanisms linking SPS and SPN to CA. Previous research has consistently indicated a strong association between LS and social support, emphasizing the key role of LS as a mediating factor that contributes to improving psychological health (Yildirim et al., 2025). Research has further suggested that individuals with higher levels of LS tend to report more positive attitudes toward their own aging, indicating that broader evaluations of life circumstances may shape perceptions of the aging process(Velaithan et al., 2024). In our study, we focused on older adults, a group characterized by complex psychological and social processes, making it essential to explore how SPS and SPN may influence ATOA. Although LS and ATOA have been independently demonstrated to exert significant effects across various psychological models, research simultaneously addressing their collective impact within an integrated framework remains limited. The significant associations among these variables provide a theoretical basis for our proposed chain mediation model. Moreover, the differences between SPS and SPN provide a new perspective on the independent variables, allowing us to distinguish the positive and negative pathways linking SPS and SPN to CA via LS and ATOA. Accordingly, we propose the following model among older adults.

**Figure 1:**
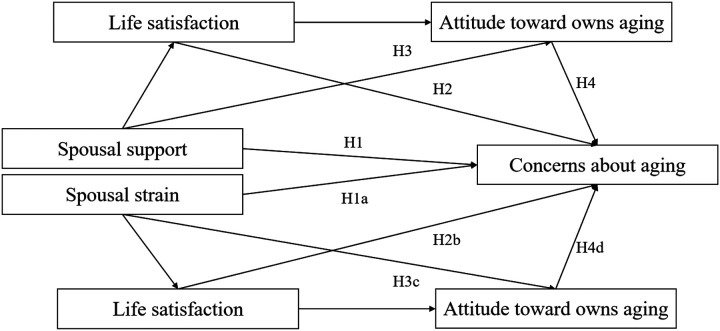
Hypothesis model Notes: Life satisfaction and attitude toward owns aging were same variable, detaching for clearer observation

The present model (Fig. 1) integrates social relationship and aging-perception perspectives to explain how spousal relationship quality may influence aging-related concerns through broader evaluations of life and aging-specific attitudes.

H4: LS and ATOA mediate the relationship between SPS and CA in a chain mediation model.

H4d: LS and ATOA mediate the relationship between SPN and CA in a chain mediation model.

## Methods

### Data Source and Participants

The data were drawn from the Health and Retirement Study (HRS), a nationally representative longitudinal panel survey sponsored by the National Institute on Aging (grant number NIA U01AG009740) and conducted by the University of Michigan. Since 1992, the HRS has followed U.S. adults aged 50 years and older through biennial interviews, included more than 20,000 respondents in each wave. The HRS employs a multistage stratified area probability sampling design to obtain a nationally representative sample of older adults in the United States. The present study used data from the psychosocial questionnaire administered in the 2022 HRS wave.

The HRS was approved by the Institutional Review Board of the University of Michigan (HUM00061128), and all participants provided written informed consent. The current study used de-identified publicly available data and was therefore exempt from additional institutional review.

The psychosocial component of the 2022 HRS included 15,856 respondents. Due to the rotating administration design of the psychosocial questionnaire, only a randomly selected subsample of respondents completed these measures in each survey wave. Based on the variables required for the present study, 4,453 participants had available data on the study variables

#### Measures

##### Spousal support

Spousal support was measured using three items adapted from the Emotional Support Measure, originally derived from the Midlife in the United States (MIDUS) survey. Participants were asked about their perceptions of emotional support from their spouse or partner. Participants responded on a 4-point Likert scale (1 = A lot, 4 = Not at all). A representative item was “How much can you open up to them if you need to talk about your worries?” These items were reverse-coded to maintain consistency in scoring and directionality across the scale. Responses were coded such that higher scores reflected greater spousal support. The scale demonstrated good internal consistency (Cronbach’s α = 0.831).

##### Spousal strain

Spousal strain was measured using four items adapted from the same Emotional Support Measure. Participants received the same introductory statement as that used for the spousal support measure and rated each item on a 4-point Likert scale (1 = A lot, 4 = Not at all). An representative item was, “How much do they let you down when you are counting on them?” Responses were coded such that higher scores reflected higher spousal strain. The measure demonstrated acceptable reliability (Cronbach’s α = 0.780).

##### Life satisfaction

Life satisfaction was assessed with five items adapted from the Satisfaction With Life Scales. Participants were asked to indicate their agreement with the following statements, “Please say how much you agree or disagree with the following statements,” and indicated their responses on a 7-point Likert scale ranging from 1 (Strongly disagree) to 7 (Strongly agree). A representative item was, “So far, I have gotten the important things I want in life.” Items were coded such that higher scores denoted greater life satisfaction. The measure showed good internal consistency (Cronbach’s α = 0.888).

##### Attitudes toward own aging

Attitudes toward own aging was assessed with eight items adapted from the HRS psychosocial questionnaire. Participants were asked to indicate their agreement with statements regarding aging experiences and perceptions, “The next statements are about the way people feel about their age and about the things that happen as they get older. Please tell us how much you agree or disagree with each statement for you personally.” and indicated their responses on a 6-point Likert scale ranging from 1 (Strongly disagree) to 6 (Strongly agree). A representative item was, “Getting older has brought with it many things that I do not like.” The items (1, 3, 7, 8) were reverse-coded to maintain consistency in scoring across the scale. Items were coded such that higher scores denoted greater positive attitudes toward own aging. The measure showed acceptable internal consistency (Cronbach’s α = 0.809).

##### Concerns about aging

Concerns about aging was assessed using five items adapted from the HRS psychosocial questionnaire. Participants rated their level of concern regarding aging-related issues, “Please rate your own level of concern about the following as you get older” and rated each item on a 4-point Likert scale (1 = Extremely concerned, 4 = Not at all concerned). An example item was, “Paying for health care expenses (e.g., co-pays, prescription drugs, uncovered expenses).” Responses were coded such that higher scores reflected greater aging-related concerns. The measure demonstrated satisfactory reliability (Cronbach’s α = 0.839).

##### Statistical analysis

Descriptive statistics and Pearson correlations among the study variables were examined using SPSS 26.0. Little’s Missing Completely at Random (MCAR) test indicated that the data were not missing completely at random (χ^2^ = 4071.94, df = 2984, *p* < 0.001). Therefore, missing data were handled using full information maximum likelihood (FIML) estimation in subsequent analyses.

Confirmatory factor analysis (CFA) in Mplus 8.3 was used to test the construct validity with robust maximum likelihood (MLR). Model fit was considered acceptable when the comparative fit index (CFI) and Tucker-Lewis index (TLI) exceeded 0.90 and the root mean square error of approximation (RMSEA) was below 0.08 (Hu & Bentler, 1999).

Mediation analyses were conducted using the bootstrap procedure as recommended by Hayes. Unlike the Sobel test, the bootstrap method empirically estimates the sampling distribution, avoiding the assumptions of normality and offering greater statistical power with reduced Type I error (Hayes, 2009). We used maximum likelihood (ML) and employed 5,000 bootstrap resamples to obtain bias-corrected 95% confidence intervals for the mediation effects. All mediation models and fit evaluations were carried out in Mplus 8.3.

## Results

### Discrimination validity check

CFA was conducted to evaluate whether the measurement model of the adopted scales in the present sample of older adults. The model was estimated using MLR estimator. To preserve the original measurement structure, all items were retained and no item parceling or post hoc model modifications were applied. The hypothesized measurement model demonstrated marginal-to-acceptable fit to the data, χ^2^(265) = 3837.33, *p* < 0.001, RMSEA = 0.055, CFI = 0.893, TLI = 0.879, SRMR = 0.046. Although the chi-square statistic was significant, which is common in large samples and complex models, the absolute fit indices suggested acceptable overall model fit.

### Sample Characteristics and Descriptive Correlations

Participant characteristics are presented in Table 1. The sample comprised 4,453 older adults (Mage = 69.27, SD = 10.44), of whom 60.03% were female. Most participants were White/Caucasian and had at least a high school education. Descriptive statistics for spousal relationship and aging-related variables are also presented in Table 2.

Table 2 presents the descriptive statistics and Pearson correlations among the study variables. SPS was negatively associated with SPN and CA, and positively correlated with LS and ATOA, whereas SPN was negatively associated with LS and ATOA and positively correlated with CA. In addition, LS was positively associated with ATOA and negatively associated with CA, whereas ATOA was negatively correlated with CA. Overall, the observed correlations were consistent with the hypothesized relationships and provided preliminary support for the mediation model.

### Test of the hypothesized serial mediation model

Table 3 presents the standardized direct, indirect, and total effects based on 5,000 bootstrap resamples. The total effects of SPS (β = 0.108, 95% CI [0.037, 0.174]) and SPN (β = 0.071, 95% CI [0.002, 0.145]) on CA were significant. However, the direct effects of SPS (β = 0.056, 95% CI [−0.012, 0.121]) and SPN (β = 0.010, 95% CI [−0.058, 0.085]) were non-significant, whereas the total indirect effects were significant, indicating indirect-only mediation effects.

Regarding specific indirect pathways, the LS-mediated pathways from SPS and SPN to CA were non-significant. In contrast, the indirect pathway from SPS to CA through ATOA was significant, whereas the corresponding pathway from SPN through ATOA did not reach significance.

Significant serial mediation effects were observed for both SPS and SPN. SPS exerted an indirect effect on CA through the sequential pathway of LS and ATOA (β = 0.023, 95% CI [0.014, 0.036]). Likewise, the serial pathway from SPN to CA via LS and ATOA was significant (β = 0.036, 95% CI [0.024, 0.052]).

## Discussion

In this study, we sought to identify and examine the mechanisms underlying two pathways linking SPS and SPN with CA among older adults. SPS and SPN were significantly associated with CA through indirect pathways, whereas their direct effects did not reach significance. The findings of this study suggest that: (1) Higher levels of SPS were significantly associated with lower levels of CA through a serial mediation pathway involving LS and ATOA. (2) Higher SPN was significantly associated with greater CA through a serial mediation pathway involving LS and ATOA.

In recent decades, there has been a great deal of research examining the role of SPS in older adults across different psychological mechanisms. While prior research has linked variables such as loneliness, depressive symptoms, subjective well-being, and health to SPS, a review of the literature indicates a notable paucity of studies that have concurrently explored the relationship between spousal relationship quality and CA with explicit attention to the mediating processes involved. Cobb (1976) first introduced and systematically articulated the idea that social support could serve as a buffering factor within health-related pathways, thereby laying the foundation for understanding its significance in attenuating the negative consequences of poor health. Along these lines, Berkman and Syme (1979) conducted an empirical study demonstrating that individuals with social support showed significantly better long-term outcomes. The theoretical foundation of SPS can be traced to the view that intimate relationships serve as a stable and enduring source of support throughout the life course (Kahn & Antonucci, 1980). These findings provide a theoretical basis for understanding potential pathways linking SPS and CA. Recent research has identified several correlations between emotional support and negative psychological outcomes. A longitudinal study using HRS data found that SPS was positively associated with five indicators of mental health, whereas SPN was positively related to three indicators of negative mental health, highlighting subtle inconsistencies in the effects of support and strain under specific circumstances (de la Rosa et al., 2025). Studies have confirmed that marital strain in older adults contributes to declines in subjective well-being, exacerbation of depressive symptoms, and intensification of loneliness (Ermer & Proulx, 2022). However, previous studies have not examined the relationship between SPS or SPN and CA, nor have they used mediation analysis or structural equation modeling to explore the underlying psychological mechanisms. Rather, regression approaches were predominantly used to examine the broad range of influences attributable to SPS and SPN. Our study, in contrast, adopts a perspective centered on CA, an increasingly important psychological concern among older adults. Notably, when investigating the associations between SPS or SPN and CA, it is essential to consider the distinction between direct and indirect effects, particularly in the context of complex interactions among psychological processes.

In the present study, both SPS and SPN were significantly associated with CA. In general, according to the positive–negative effect model, support and strain are typically conceptualized as parallel pathways with distinct mediating mechanisms (Ingersoll-Dayton et al., 1997). For example, higher levels of SPS, similar to emotional support, are linked to more favorable health outcomes, whereas higher SPN exerts opposite effects (Reblin & Uchino, 2008). While the overall pattern of total effects observed in the present study is consistent with prior research, the indirect pathways reveal nuanced differences in the underlying psychological mechanisms. Because earlier studies rarely tested complex mediation pathways, direct comparisons with our results are limited. For example, research exploring the links between spousal relationship quality and various health indicators, as well as studies investigating the determinants of spousal relationship quality, can offer valuable insights into these mechanisms (Hoppmann & Gerstorf, 2009). Moreover, the inclusion of additional mediators may reveal additional indirect pathways linking SPS or SPN to CA, particularly through broader psychosocial and behavioral processes.

The findings of this study suggest that SPS is significantly related to CA through ATOA and the serial pathway involving LS and ATOA. The chain mediation model further indicated that SPS is significantly related to LS, which in turn is associated with ATOA and ultimately links to CA. Notably, LS as an independent mediator did not exhibit a significant indirect association. These findings are consistent with studies indicating a significant association between SPS and positive mental factors (Biehle & Mickelson, 2012; Priem & Solomon, 2015). The primary difference between SPS and SPN appears to involve the mediator ATOA. The indirect pathway from SPN to CA through ATOA did not reach significance. These findings provide evidence that the relationships between SPS, SPN, and CA were primarily transmitted through indirect pathways. This mediating mechanism involves LS, ATOA, and potentially additional mechanisms that have yet to be empirically examined. Concerning the mediator ATOA, the non-significant indirect path from SPN to CA through ATOA may result from the distinct psychological nature of positive versus negative relational origins. This finding highlights nuanced distinctions between SPS and SPN. While both exhibited significant total associations with CA, the mediating effects differed across pathways. Moreover, the results remind us that although SPS and SPN may originate from similar measurement items, their opposite directions may shape distinct psychological pathways, making it essential to identify the underlying mechanisms in their relationships.

Previous research findings are largely consistent with our results. For example, there is a significant association between spousal well-being and LS (Powdthavee, 2009; Carr et al., 2014). Conversely, negative emotional states in one spouse, including stress, tension, and fatigue, exhibit significant positive correlations with the partner’s negative emotional condition (Westman & Etzion, 1995). This may partly explain the reciprocal influence of emotional states between spouses. Regarding ATOA, older adults with a more positive ATOA tend to report higher LS, better self-rated health, and lower levels of anxiety and depression (Bryant et al., 2012). Previous research has demonstrated that ATOA serves as a robust predictor of health-promoting behaviors among older adults (Korkmaz Aslan et al., 2017). Our chain mediation model highlights CA as a complex psychological outcome shaped by multiple risk-related factors, and negative attitude toward one’s own aging may ultimately lead to more severe CA. Fear of aging among older individuals seems closely related to the manner in which they engage with later life and to their anticipations regarding the future (Rupprecht et al., 2022). Prior research on spousal emotional dynamics and mental health corroborates our findings, demonstrating that the emotional conditions of emotional conditions within spousal relationships are significantly associated with mental health outcomes among older adults.

The present study is subject to certain limitations, primarily related to the cross-sectional design, which precludes causal inference. Second, to extend the present findings, it is necessary to use a appropriate designs to clarify the temporal and potentially causal relationships among SPS, SPN, and CA. This will enable us to develop a more comprehensive understanding of the interrelationships among SPS, SPN, LS, ATOA, and CA.

Although a key strength of our study is the use of comprehensive HRS data, covering a nationally representative sample of U.S. older adults, the study sample was predominantly White, with White participants accounting for 71.94% of the sample, which may affect the generalizability and external validity of the conclusions. Moreover, because the sample includes individuals aged 50 years and older, these findings may not generalize to younger populations.

Although our results suggest significant psychological mechanisms, the insufficient consideration of potential confounding factors remains a limitation, as only basic demographic variables were included. In large-scale studies, even small associations may reach statistical significance, which may affect the estimated path coefficients and limit the robustness of the findings.

Despite these limitations, our results underscore the importance of SPS and SPN in shaping life satisfaction, attitudes toward own aging, and aging-related concerns. While the pathways from SPS to CA and from SPN to CA shared certain mediating features, meaningful differences were also observed across pathways. These findings emphasize the need for greater attention to and interventions targeting spousal relationship quality and emotional dynamics among older adults.

## Figures and Tables

**Figure 2:**
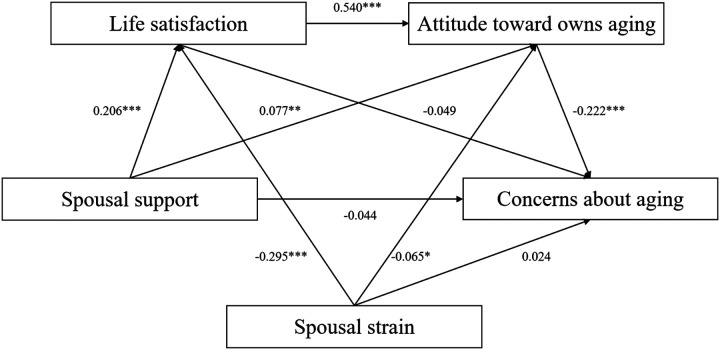
The chain mediation model of the spousal support and strain on concerns about aging

**Table 1:** Participant Characteristics (N = 4453)

Characteristic	No. (%)	*M* (*SD*)
Sociodemographic		
Age		69.27 (10.44)
≤50 years	74 (1.66)	
50–64 years	1659 (37.26)	
≥65 years	2720 (61.08)	
Gender		0.60 (0.49)
Male	1780 (39.97)	
Female	2673 (60.03)	
Race/ethnicity		
White/Caucasian	2988 (67.10)	
Black or Africa American	902 (20.26)	
Other	452 (10.15)	
Not obtained	111 (2.49)	
Highest degree of education		3.06 (2.23)
No degree	532 (11.95)	
GED	210 (4.72)	
High School diploma	1692 (38.00)	
Two year college degree	307 (6.89)	
Four year college degree	797 (17.90)	
Master degree	483 (10.85)	
Professional degree (Ph.D., M.D., J.D.)	305 (6.85)	
Spousal relationship quality		
SPS	2846	10.35 (2.08)
SPN	2838	7.65 (2.70)
Psychological well-being and aging perceptions		
LS	4399	25.26 (7.47)
ATOA	4384	31.70 (8.10)
CA	4375	13.36 (3.65)

Notes: Gender, degree were categorical variables coded as: gender (1 = male, 2 = female), degree (1 = No degree; 2 = GED; 3 = High school diploma; 4 = Two year college degree; 5 = Four year college degree; 6 = Master degree; 7 = Professional degree (Ph.D., M.D., J.D.). Valid sample sizes varied across variables due to missing data.

**Table 2:** Means, standard deviations, and correlations among study variables

Variable	Mean (SD)	1.	2.	3.	4.	5.	6.	7.	8.
1. Gender	1.52 (0.50)	-							
2. Age	71.01 (10.01)	−0.31*	-						
3. Degree	3.21 (2.21)	−0.07***	−0.23***	-					
4. SPS	10.35 (2.08)	−0.15***	0.42*	0.07***	-				
5. SPN	7.65 (2.70)	−0.01	−0.07***	−0.00	−0.46***	-			
6. LS	25.26 (7.47)	−0.01	0.09***	0.00	0.32***	−0.34***	-		
7. ATOA	31.70 (8.10)	−0.00	−0.16***	0.12***	0.24***	−0.25***	0.47***	-	
8. CA	13.36 (3.65)	0.11***	−0.01	0.01	−0.12***	0.11***	−0.20***	−0.26***	-

Notes: Gender and degree were coded as: gender (1 = male, 2 = female), degree (1 = No degree; 2 = GED; 3 = High school diploma; 4 = Two year college degree; 5 = Four year college degree; 6 = Master degree; 7 = Professional degree (Ph.D., M.D., J.D.).

* *p* < 0.05,

** *p* < 0 .01,

*** *p* < 0.001.

**Table 3:** Standardized direct, indirect, and total effects of SPS and SPN on CA

Predictor	Effect	β	Boot SE	95% CI [LL,UL]	Proportion mediated
SPS	Total effect	−0.099	0.033	[−0.164,−0.033]	-
	Direct effect	−0.044	0.033	[−0.109, 0.019]	-
	Total indirect effect	−0.055	0.006	[−0.076,−0.038]	55.56%
	LS → CA	−0.010	0.006	[−0.023, 0.000]	-
	ATOA → CA	−0.018	0.007	[−0.035,−0.005]	18.18%
	LS → ATOA → CA	−0.027	0.005	[−0.038,−0.018]	27.27%
SPN	Total effect	0.092	0.034	[0.026, 0.159]	-
	Direct effect	0.024	0.035	[−0.043, 0.094]	-
	Total indirect effect	0.068	0.011	[0.048, 0.090]	73.91%
	LS → CA	0.015	0.008	[0.000, 0.031]	-
	ATOA → CA	0.016	0.008	[0.001, 0.031]	17.39%
	LS → ATOA → CA	0.038	0.006	[0.027, 0.050]	41.30%

Notes: All estimates are standardized coefficients (β). Bootstrap standard errors and 95% bias-corrected confidence intervals were based on 5,000 bootstrap resample.

Notes: Life satisfaction and attitude toward owns aging were same variable, detaching for clearer observation

## Data Availability

The de-identified dataset and study materials are available through the Health and Retirement Study (HRS) website and the National Archive of Computerized Data on Aging (NACDA).
